# Diabetic Neuropathy Is Related to Rhinencephalon Degeneration in Adults With Type 1 Diabetes

**DOI:** 10.1155/2024/6359972

**Published:** 2024-10-07

**Authors:** Maciej Chudzinski, Katarzyna Karmelita-Katulska, Anna Duda-Sobczak, Tatiana Fijalkowska-Ratajczak, Jakub Kopec, Michal Michalak, Dorota Zozulinska-Ziolkiewicz, Aleksandra Araszkiewicz

**Affiliations:** ^1^Department of Internal Medicine and Diabetology, Poznan University of Medical Sciences, Poznan, Poland; ^2^Department of General Radiology and Neuroradiology, Poznan University of Medical Sciences, Poznan, Poland; ^3^Department of Otolaryngology, Raszeja City Hospital, Poznan, Poland; ^4^Department of Computer Sciences and Statistics, Poznan University of Medical Sciences, Poznan, Poland

**Keywords:** central neuropathy, diabetic peripheral neuropathy, olfactory function, rhinencephalon, type 1 diabetes

## Abstract

**Aims:** We aimed to assess neurodegenerative changes in the rhinencephalon via magnetic resonance imaging (MRI) and relate it to olfactory function and diabetic peripheral neuropathy (DPN) in adults with type 1 diabetes (T1D).

**Materials and Methods:** Individuals aged 18–65 with T1D duration over 10 years and control healthy subjects underwent olfactory assessment using Sniffin'Sticks and brain MRI to assess volumetric measurements of the olfactory bulbs and piriform cortex thickness.

**Results:** 32 T1D (24 males) aged 43.5 years (IQR: 37.0–48), diabetes duration 24.5 years (IQR: 20.5–27.0), and A1C 7.95% (IQR: 7.4–8.4) were assessed. The control group consisted of 6 healthy adults (4 males) aged 41.0 years (IQR: 36.0–48.0). Significantly lower olfactory test results in TDI (threshold-differentiation-identification) (31.5 (IQR: 28.7–33.6) vs. 34.1 (IQR: 33.2–37.2), *p* = 0.02) were obtained in the T1D as compared to the controls. Summarized olfactory bulb (OB) volumes and thickness of the left pyriform cortex were significantly smaller in T1D than in controls (65.8 mm^3^ (IQR: 57.9–71.7) vs. 75.8 mm^3^ (IQR: 74.8–76.7); *p* = 0.0005 and 3.1 mm (IQR: 2.7–3.4) vs. 3.6 mm (IQR: 3.5–4.1); p =0.02). Patients with DPN had significantly smaller OB volumes than patients without DPN (58.1 mm^3^ (IQR: 54.0–70.9) vs. 69.8 mm^3^ (IQR: 65.0–72.2); *p* = 0.02). Tobacco smoking (*β*: −7.89; *p* = 0.013) and DPN (*β*:−7.02; *p* = 0.015) proved to be independent predictors of OB volume.

**Conclusions:** In adults with a long history of T1D, olfactory function and structures are impaired. The presence of diabetic neuropathy and ongoing smoking addiction might be considered predictors of the degradation of rhinencephalon structures in people with T1D.

## 1. Introduction

Diabetic peripheral neuropathy (DPN) is the most common complication of diabetes, comprising approximately 50% of patients, expressed mainly as an impaired sense of touch, pain, and vibration. Approximately 12% of patients with DPN present with its painful form, dominantly with paresthesia [1, 2]. Central nervous system neuropathy has been recently intensively investigated and brain imaging showed alterations in many central structures including sensory fields in the neocortex [3, 4]. Cranial nerves, comprising olfactory nerves, are structures closely related to the brain that are relatively easily accessible for physical examination. Olfactory impairment and its relation to DPN has been previously shown in patients with type 1 diabetes (T1D) [5]. Several research studies showing morphological changes in the central nervous system in subjects with diabetes in magnetic resonance imaging (MRI) have been published. Reske-Nielsen and Lundbaek in their postmortem studies performed in patients with long-lasting diabetes and a history of DPN demonstrated atrophy in the areas of the dorsal column of the spinal cord [6]. Eaton et al. using MRI showed a significant reduction of the cross-section area in the cervical spinal cord in patients with diabetes and DPN compared to healthy controls [7]. Wilkinson et al. demonstrated numerous morphological changes obtained by MRI in patients with diabetes, namely, atrophy in the spinal cord and areas of the sensory cortex of the brain [8]. Similar changes were observed in the somatosensory perception areas in the brain [3, 4]. According to Selvarajah et al., MRI is the best tool to assess the extent of brain involvement in patients with diabetes. Thanks to its noninvasive and radiationless character, it is possible to perform it repeatedly and safely in the same patient and therefore to follow the dynamics of changes in time [3]. Previous studies showed impaired olfactory threshold, odor differentiation, and identification in diabetes [9–11]; however, the mechanisms remain unclear. Some researchers attributed it to chronic micro and macroangiopathic complications or diabetic neuropathy itself [5, 12]. Interesting aspects were raised by Brady et al. who showed an association between the presence of neuropathic pain and olfactory dysfunction [13].

This study is aimed at assessing potential neurodegenerative changes in the rhinencephalon via MRI and relating it to the olfactory function and DPN in adults with T1D.

## 2. Materials and Methods

### 2.1. Study Procedures

The study included adult patients (18–65 years) with T1D duration > 10 years, who were consecutively under the care of the Department of Internal Medicine and Diabetology Poznan University of Medical Sciences (PUMS) from August 2019 up to May 2022. All participants were treated with multiple daily injections (MDI) of insulin therapy. Considering the plasticity of human olfactory apparatus and numerous conditions altering the anatomy and functionality of the sense of smell, the following exclusion criteria have been applied: diagnosis of neurodegenerative disorders (i.e., Alzheimer's, Parkinson's, and Huntington's disease and multiple sclerosis) and neuropsychological diseases including dementia, depression, schizophrenia, and bipolar disease. History of stroke, head trauma, serious anatomical alteration to the nasal cavities, upper respiratory tract infections, chronic noninfectious diseases of the nose and paranasal sinuses, allergic rhinitis, and vasculitis were also exclusions.

In further analyses, the study group was divided according to the presence of DPN. The control group consisted of healthy (diabetes-free) subjects of similar age, who did not present any of the exclusion criteria.

The project was approved by the local ethics committee in PUMS, decision number 719/19 from June 19, 2019, with later changes in 208/2021 from March 11, 2021. All participants provided informed, written consent.

All participants filled out a questionnaire considering their medical history, medications, smoking habits, severe hypoglycemia, and ketoacidosis episodes in the past 12 months. Patients underwent anthropometric measurements and full ear, nose, and throat (ENT) examination, including nasal endoscopy, by two experienced otolaryngologists, to exclude any possible preexisting condition compromising olfaction. Every patient was also subjected to Mini Mental State Examination (MMSE) to exclude potentially undiagnosed, underlying dementia (permission from Psychological Assessment Resources (PAR) was granted to use this scale in the research, licensed version from Polish Psychological Association).

Laboratory tests were performed on the Cobas 6000 apparatus.

### 2.2. Outcome Definition

Peripheral neuropathy was assessed based on symptoms and signs. Diagnosis of DPN was based on Toronto's definition of probable peripheral neuropathy as the presence of two or more of the following: the presence of symptoms, lack of the ankle reflex, and impaired sensation of touch, temperature, and/or vibration. Symptoms were assessed based on the medical history. On physical examination, the ankle reflex and peripheral sensation (temperature using a TipTherm, touch using a 10 g monofilament, vibration using a 128 Hz tuning fork) were evaluated [14]. To assess possible painful neuropathy symptoms the DN4 (Douleur Neuropathique en 4 Questions) questionnaire was used—a score of ≥ 4 indicated the presence of painful neuropathy [15].

The olfactory function was evaluated using a standardized testing battery of Sniffin'Sticks. The battery consists of three separate tests evaluating the olfactory threshold and ability to differentiate and identify odors. The result is presented as a sum of all 3 tests in the form of a TDI (threshold-differentiation-identification) index. Patients were asked to refrain from eating and smoking at least 1 h before the examination. The threshold test consists of 48 sticks arranged in 16 groups of three. Every trio contains one n-butanol-infused stick in gradually increasing concentration. The test is performed with the eyes covered. The patient is presented with three sticks in rounds, gradually increasing the concentration of the target odor until it is identified correctly twice. Next, the trios are presented in decreasing concentration order until the target odor is no longer identified. The number of altering points is then noted. The test is continued until seven full alterations are completed. The average of the last four tests indicates the olfactory threshold. The lower the numerical value, the higher the olfactory threshold. The differentiation test consists of 48 sticks grouped in 16 trios. The patient is presented with each of the three sticks, one by one. Patients indicate the stick containing odor that is different from the other two. In the identification test, 16 odors are presented consecutively and the patient identifies the odor by picking one of four possible answers. The results are summarized. TDI of ≤ 30.5 indicates hyposmia [16].

### 2.3. MRI

**Figure 1:**
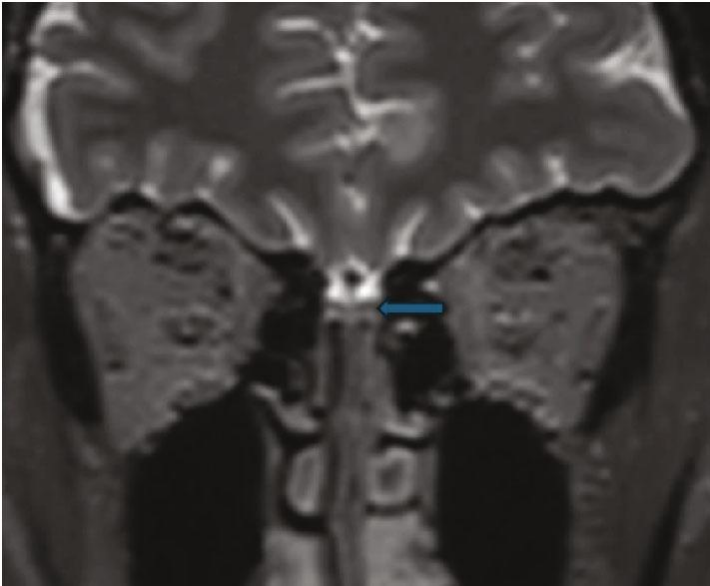
Coronal T2-weighted images (arrow) of olfactory bulb left.

**Figure 2:**
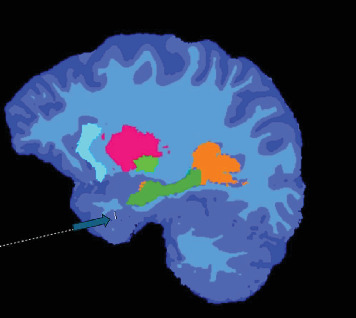
Coronal T1-weighted MR-images depicts a segmentation of the piriform cortex.

Noncontrast MRI of the head was performed to examine the morphology of the rhinencephalon. T1 and T2 sequences with 3D MPRANGE sequences were obtained to measure total intracranial volume, olfactory bulbs' (OB) volume, and the piriform cortex's (PCo) thickness (Figures 1 and 2). For the quantitative, volumetric analysis FreeSurfer vs. 12.23 and Syngovia Neurology 3D Siemens Erlangen software were used. The MRI scan was performed on a 3T Magnetom Skyra Siemens Erlangen device. All of the measurements were conducted by one experienced radiologist.

### 2.4. Statistical Analyses

Statistical analysis was performed using STATISTICA 13 PL (StatSoft, Tulsa, Oklahoma, United States) software and STATA 17 (StataCorp. 2021. Stata Statistical Software: Release 17. College Station, TX: StataCorp LLC). Due to numerous deviations from the normal distribution of data in the Shapiro–Wilk test, the numerical characteristics of the groups were presented using medians and interquartile range (IQR). The Mann–Whitney *U* test was used for comparative analyses between T1D and control groups and between groups with and without DPN assessing differences in the size of rhinencephalon structures and olfactory test results. The Kruskall–Wallis test for multiple subgroup analyses (ANOVA for nonparametric variables) along with post hoc analysis tests—multiple comparisons of mean ranks (Dunn's test)—was performed to determine whether any differences in rhinencephalon structures and olfactory test results between patients with DPN and without DPN and the controls were present. Due to the lack of available literature data regarding the population of patients with T1D and, as a result, the inability to estimate the size of expected differences between groups, no preliminary calculations of the necessary sample size were made. The relationship between the studied variables was assessed using Spearman's correlation test. A stepwise multiple linear (sigma restricted) regression model was performed to evaluate the impact of the studied variables on the size of the OBs. The model included factors significantly associated with volumes' parameters in the comparative and correlation analyses: TDI, MMSE, and glycated hemoglobin A1c percentage as quantitative predictors. Sex, smoking status, and the presence of neuropathy are qualitative predictors. Since the obtained data did not meet the required assumptions, such as normality of residuals, homoscedasticity, or linearity, we applied the bootstrapping method to assess the stability and reliability of the obtained results. Additionally, we denoted Cliff's delta as the effect size measure for compared variables (by the nonparametric Mann–Whitney test). Cliff's delta can be positive or negative. A positive delta indicates that the first group tends to have higher ranks than the second group. A negative delta indicates that the second group tends to have higher ranks than the first group. Cliff's delta values around 0.1, 0.3, and 0.5 denote small, medium, and large effects, respectively. The two one-sided tests (TOST) method was used for testing equivalence between two groups to justify the relevant difference between groups. If both one-sided tests reject their respective null hypotheses, we can conclude that the difference between the groups is statistically equivalent otherwise the equivalence cannot be concluded. As the delta margin, we choose an arbitrary 75% of the assessed effect size. For the calculations we used a two-sample rank-sum test for stochastic equivalence “tostranksum” and as effect size the Hodges–Lehmann shift estimate “npshift”.

All tests were performed at the significance level *α* = 0.05.

## 3. Results

### 3.1. Group Characteristics

The study group initially involved 41 patients, 2 were excluded due to laryngological causes (septum ulcers and perforation), and 7 withdrew their consent due to the ongoing COVID-19 pandemic. The final 32 patients (24 males) were included in the statistical analysis. The median age in the study group was 43.5 years (IQR: 37.0–48.5), diabetes duration 24.5 years (IQR: 20.5–27.0), and A1c 7.95% (IQR: 7.4–8.4). A median daily insulin dose was 0.46 units/kg of body weight/24 h (IQR: 0.38–0.6). Two patients had a documented history of severe hypoglycemia in the past 12 months, and no one had a ketoacidosis episode in the past 12 months. We assessed the presence of chronic microvascular complications of diabetes: DPN was present in 15 cases (46.9%), cardiac autonomic neuropathy in 8 cases (25%), diabetic kidney disease in 7 cases (21.9%), and retinopathy in 16 cases (50%). Concomitant diseases in the study group included dyslipidemia (18 cases), hypertension (13 cases), obesity and hypothyroidism (5 cases), coronary disease (2 cases), asthma (1 case), coeliac disease (1 case), and chronic hepatitis C (1 case). The control group initially comprised 10 age- and sex-matched, healthy (diabetes-free) subjects; 1 was disqualified due to a laryngological defect (nasal polyps) and 3 due to COVID-19 infection before the MRI. Finally, 6 subjects were included in the statistical analysis. Group characteristics are presented in Table 1. The study group was further divided according to the presence of DPN and then according to painful or painless DPN. Fifteen patients were diagnosed with DPN, and among them, 8 had painful neuropathy.

### 3.2. Olfactory Test Results

The study group performed statistically worse in the olfactory testing (expressed as TDI) than the control group: 31.6 (IQR 28.75–33.6) versus 34.1 (IQR: 33.25–37.25), *p* = 0.02. Patients with diabetes had significantly higher olfactory thresholds than the controls: 7.0 (IQR: 6.5–8.0) versus 8.5 (IQR: 8.0–9.0) *p* = 0.049. Patients with DPN showed statistically lower TDI results: 31.6 (IQR: 26.0–37.25) versus 34.1 (IQR: 33.25–37.25), *p* = 0.04 than the controls. However, there were no statistically significant differences in the olfactory performance between patients with DPN and those without this complication. See Table 1 and Figure S1.

### 3.3. Structural Rhinencephalon Measurements

The MRI showed a statistically smaller summarized volume of the OB in patients with T1D as compared to the controls 65.8 (IQR: 57.95–71.7) versus 75.8 (IQR: 74.8–76.7) mm^3^, *p* = 0.0005, also separately volumes of the right 33.4 (IQR: 28.6–36.2) versus 37.5 (IQR: 36.9–38.8) mm^3^, *p* = 0.001 and left 32.6 (IQR: 29.1–35.4) versus 37.95 (IQR: 37.2–38.8) mm^3^, *p* = 0.0006 OBs were statistically smaller. In addition, left PCo thickness was statistically smaller in the study group than in the controls: 3.1 (IQR: 2.8–3.4) versus 3.7 (IQR: 3.6–4.1) mm *p* = 0.02. No statistical differences were found in the thickness of the right PCo.

**Figure 3:**
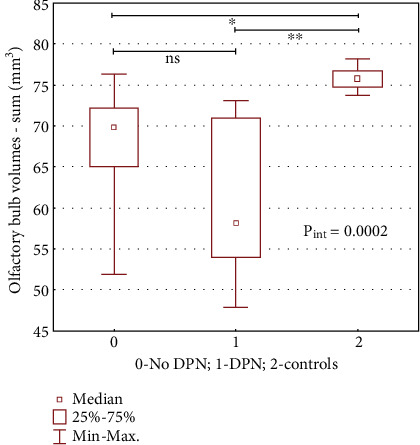
Olfactory bulb volumes (sum) in patients with Type 1 diabetes with DPN and without DPN and in healthy controls. Abbreviations: DPN, diabetic peripheral neuropathy; ns, not significant. Kruskall–Wallis test. *P*_interaction_ (*P*_int_) < 0.05 indicates that at least one subgroup result differs significantly. *p* value is represented by an asterisk ⁣^∗^*p* < 0.05; ⁣^∗∗^*p* < 0.01;

Subjects with T1D and DPN proved to have smaller summarized OB volumes than subjects without DPN: 58.1 (IQR: 54.01–70.09) versus 69.8 (IQR: 65.02–72.2) mm^3^, *p* = 0.02. Moreover, the right and left OB volumes were also smaller than in patients without DPN (29.3 (IQR: 26.9–34.7) vs. 34.5 (IQR: 32.7–36.7) mm^3^, *p* = 0.02 and 29.5 (IQR: 27.1–35.2) vs. 34.6 (IQR: 31.8–35.6) mm^3^, *p* = 0.01, respectively). In patients with DPN as compared to the controls, only the thickness of the right PCo did not show a statistically significant difference. In subjects without DPN, only the sum of OB volumes, and the volume of the left OB separately was significantly smaller than in the controls. Differences in rhinencephalon structures between groups with and without DPN proved to be statistically insignificant using this test. See Table 1 and Figure 3.

### 3.4. Painful DPN

Patients with T1D were subjected to DN4 examination to determine whether painful neuropathy is present. Out of 32 patients, 8 fulfilled the criteria of painful neuropathy (≥ 4 points). Patients with painful neuropathy had better results in olfactory testing than those with painless DPN; however, the difference was not statistically significant. Summarized OB volumes, separately right and left OB volume, and the thickness of the PCo in patients with painful neuropathy were smaller than in patients with painless DPN, and this comparison also did not reach statistical significance. See Table S1.

### 3.5. Clinical Characteristics and Olfactory Function in Relation to Rhinencephalon Measurements

The differences in the median daily dose of insulin between groups with neuropathy and without neuropathy proved to be statistically insignificant: 0.42 (IQR: 0.37–0.57) versus 0.56 (IQR: 0.44–0.63) units/kg of body weight/24 h, *p* = 0.08. In addition, no statistically significant differences were found in the median daily dose of insulin between groups with normosmia and hyposmia: 0.44 (IQR: 0.38–0.57) versus 0.53 (0.38–0.62) units/kg of body weight 24 h, *p* = 0.2. We found no statistically significant correlations between the daily dose of insulin and rhinencephalon measurements and olfactory test scores.

All subjects underwent MMSE to exclude the possible influence of dementia. Patients with DPN had significantly lower results in MMSE than patients without this complication: 28 (IQR: 26–29) versus 29 (IQR: 28–30); *p* = 0.03. We found a significant positive correlation between the result of the MMSE and odor differentiation test (RS = 0.41; *p* = 0.02). No significant correlation between the MMSE result and other olfactory test results or rhinencephalon morphometry was found.

We evaluated correlations between rhinencephalon structure measurements and olfactory test results in the whole group subjected to MRI (study group and controls; *n* = 38). We found that summarized OB volumes (RS = 0.32; *p* = 0.048), left OB volume (RS = 0.33; *p* = 0.04), as well as right (RS = 0.36; *p* = 0.03), and left (RS = 0.43; *p* = 0.006) PCo thicknesses were positively correlated with the TDI result. Similar correlations were not found in the diabetes group separately. See Table S2.

We fitted linear regression models including quantitative and qualitative predictors to analyze the influence of correlated variables on the volume of OB in the group of patients with T1D. The models included TDI, MMSE, and A1c percentage as quantitative predictors. Sex, smoking status, and the presence of neuropathy were chosen as qualitative predictors. In our models, tobacco smoking (*β* = −7.89; *p* = 0.013) and the presence of DPN (*β* = −7.02; *p* = 0.015) were independently associated with the volume of OBs in patients with T1D. See Table S3. Trying to build the multiple regression model, we performed using a stepwise procedure; DPN was the only significant factor for OB volume. For this purpose, we generated multiple bootstrap samples (*n* = 1000) and estimated the distribution of the estimators, which allowed for the determination of their confidence intervals. Both tobacco smoking and DPN remained significant predictors of OB volume. The bootstrap confidence intervals were as follows: for tobacco smoking, *β* = −7.89 with a 95% CI (−14.19, −1.58), and for DPN, *β* = −7.02 with a 95% CI (−12.42, −1.61).

Effect sizes for the Mann–Whitney test–Cliff's delta, for nonparametric comparisons of rhinencephalon structures and olfactory test results are presented in Table S4. Equivalence was not proven for most parameters (Table S5).

## 4. Discussion

### 4.1. Main Findings

We found that patients with T1D perform worse in olfactory testing and have smaller central olfactory structures than healthy controls. The presence of diabetic neuropathy and ongoing smoking addiction are important determinants of the degradation of rhinencephalon structures in people with T1D.

### 4.2. Olfactory Testing in T1D and Controls

In the general population, olfactory dysfunction ranges between 2.7% and 24.5%, depending on the method of examination and population studied. Previous research has shown that olfactory function in the general population deteriorates with age and is significantly worse in men [17], and in populations aged over 80 years, olfactory deficits reach up to 62.5% [18]. The numerous variables altering the sense of smell make it particularly difficult to assess the influence of single factors. To isolate the impact of neuropathy on the sense of smell, we accepted a wide range of exclusion criteria as mentioned above; unfortunately, due to the unexpected outbreak of the COVID-19 pandemic at the time of the study, with persistent olfactory dysfunction as one of its main symptoms, the history of COVID-19 was accepted as an additional exclusion criterion.

In this study, we showed that patients with T1D had significantly lower olfactory test results by means of Sniffin'Sticks, expressed as TDI, than healthy controls. This finding is consistent with other studies [9–11, 19]. In our previous studies, patients with T1D and DPN had worse odor identification test results than patients without this complication [5]. Available studies use different olfactory testing methods, many of them are nonstandardized, and the results are often inconsistent. The use of Sniffin'Sticks was dictated by its versatility in the assessment of various aspects of olfaction and its growing presence in modern research. Olfactory testing may, however, be disturbed by numerous conditions. The ambient odor in the room, using various perfumes, cosmetics and laundry detergents, recent meals, and even surrounding temperature may influence the final result. The limitations of Sniffin'Sticks also include the durability of sticks and infused odors that weaken over time. To limit possible errors and inconsistency in performing the examination, only one researcher was designated for the task. The battery consists of three separate tests, two of which (the differentiation and identification test) require properly functioning cognitive abilities like memory, attention, and association processes. The 18–65 age range was applied to avoid the possible influence of cognitive dysfunction. Our results (lower TDI and higher olfactory threshold) suggest defects in the ability to detect olfactory particles rather than processing the information they bring. Therefore, we might suppose that patients with T1D show disturbances in the function of the lower, more peripheral olfactory structures like olfactory epithelium and OBs rather than cortical, association areas.

### 4.3. Rhinencephalon Structures in T1D and Controls in Relation to Neuropathy

To associate the olfactory deficits with structural changes to the rhinencephalon, we performed an MRI to look for possible neurodegeneration. The structures we chose were OBs and piriform cortices (areas of the primary olfactory cortex). Extensive research shows an association between OB volumes and olfactory performance [20–22]. The OB is one of the most actively remodeling structures of the human brain, and it shows continuous neuro and synaptogenesis as a response to chemosensory stimuli during whole adult life [22]. The dependence of the OB volume on the continuous proper stimulation was the main reason behind including these structures in our study's analysis of neurodegeneration. Neuropathy might cause damage to the afferent fibers of the olfactory nerve, causing a reduction in stimulation and, subsequently, the volume of the OBs. However, this assumption is limited by the fact that olfactory nerve conductivity and structural integrity might be affected by many factors other than neuropathy. To minimize the influence of those factors, we applied extensive exclusion criteria as mentioned above.

To our knowledge, our study is first to describe the relationship between OBs and PCo size to olfactory performance in adult patients with T1D. To evaluate the influence of diabetic neuropathy on higher olfactory structures, we chose the area of the PCo, which receives axons of olfactory tract fibers. The PCo is classified as a structure of the primary olfactory cortex. Research shows that gray matter thickness in this area is associated with olfactory performance [23–25]. Increased PCo thickness was observed in students and experts of the French school of perfumery (Institute Supérieur International de la Parfumerie) as compared to the control group of subjects not related to the perfume industry [24]; also, its significant reduction was observed in patients with hyposmia and anosmia as compared to healthy controls [25, 26]. Bitter et al. showed that in patients with hyposmia as well as anosmia, the decrease in thickness of the PCo is present only in the right hemisphere [26]. Similarly, in our study, we observed smaller PCo thickness in patients with diabetes as compared to the controls; however, the reduction was significant only in the left hemisphere. The study of Bitter et al. was performed exclusively in right-handed subjects; in our study we did not consider lateralization as an exclusion criterion. Lateralization may influence the size of olfactory brain structures. Acquired results might suggest the involvement of the central nervous system in the neuropathic process. In previous studies on olfaction performed in subjects with diabetic neuropathy, olfactory deficits were related to the presence of neuropathic pain. In the study of Brady et al., decreased olfactory performance measured with Sniffin'Sticks in the olfactory threshold was found in patients with T2D and neuropathic pain as compared to the general cohort of patients with T2D. The authors suggested that decreased attention and concentration abilities due to pain might have influenced the results [13]. In the study of Mizera et al. performed on the general population, subjects with a history of chronic use of analgesics had decreased olfactory function as compared to the population without the use of analgesics [27]. Both olfactory performance and nociception are connected on the molecular and structural level because of the common presence of opioid receptors in the central nervous system including the OB. Both nociception and olfaction are processed in the phylogenetically old brain structures and as such share many common neuronal pathways [28]. In our study, although the result was not statistically significant, patients with neuropathic pain had better results in the summarized olfactory test (TDI) than patients without painful DPN which is a contradiction to Brady's findings. The small number of study participants and numerous factors influencing the olfactory performance, both external and internal, make it difficult to draw definitive conclusions. Further research in a much more numerous cohort is needed for a better understanding of the problem. Further research is needed to assess the relationship between painful and painless neuropathy and the rhinencephalon volumes. The lack of significant results might be related to the small size of subgroups.

### 4.4. Rhinencephalon Structures and Smoking

Based on the volumetric MRI analysis, we showed a positive correlation of olfactory test scores (TDI) with the summarized OBs volume, left OB volume, and PCo thickness bilaterally in the whole study cohort, however, not in a separate T1D group. These results are consistent with previously limited published data, performed in subjects without diabetes, and confirm the logical relationship between the function and structure of studied rhinencephalon areas [22]. One of the most important lifestyle factors modifying olfaction and structure of rhinencephalon is tobacco smoking, however, the published data are inconsistent. In some studies, a strong association between smoking and olfactory dysfunction is suggested [29–31] but not confirmed in others [32, 33]. Meta-analysis of cross-sectional studies on olfaction in smokers showed a 59% higher incidence of olfactory deficits in smokers compared to nonsmokers [34]. Schriever et al. demonstrated a significant reduction in OB volumes with no differences in olfactory performance simultaneously [35]. Our study involved both smokers and nonsmokers dispersed similarly in all study groups. Additionally, the multivariate linear model suggests that smoking and the presence of DPN are independently associated with the reduction in OB volumes.

### 4.5. Limitations of the Study

The small size of the study and control groups is a main limitation of our project. Increasing the number of subjects in future endeavors might improve the reliability and accuracy of presented data. In addition, excluding smokers from the study group could be more beneficial to accentuate the influence of diabetic neuropathy on olfaction as a separate factor. Furthermore, in this study, we accepted the MMSE as the only test to exclude dementia and mild cognitive impairment. A positive correlation between MMSE and differentiation test results suggests the involvement of higher cortical structures and neurodegeneration in the process of odor recognition and differentiation. There are, however, more accurate and precise tools to evaluate patients' cognitive abilities, which is a limitation of our research. More accurate diagnosis of cognitive impairment should be implemented in future studies regarding this topic.

## 5. Conclusions

In conclusion, in adults with a long history of T1D, olfactory dysfunction is associated with degenerative changes in the rhinencephalon. The presence of diabetic polyneuropathy and tobacco smoking are important determinants of the degradation of rhinencephalon structures in people with T1D.

## Figures and Tables

**Table 1:** Characteristics of the participants. Olfactory test results and structural measurements.

**Variable**	**T1D**	**Controls**	**p**	**T1D with DPN**	**T1D without DPN**	**p**
*n*	32	6		15	17	
Age (years)	43.5 (37–48.5)	41 (36–48)	0.7	48 (37–52)	42 (37–46)	0.07
Sex male/female	24/8	2/4	0.07⁣^∗^	12/3	12/5	0.4⁣^∗^
Current smoking (yes/no)	9/23	2/4	0.6⁣^∗^	5/10	4/13	0.4⁣^∗^
Hypertension (yes/no)	13/19	1/5	0.4⁣^∗^	8/7	5/12	0.2⁣^∗^
CAN (yes/no)	8/24			7/8	1/16	0.01^∗^
DR (yes/no)	16/16			10/5	6/11	0.07⁣^∗^
DKD (yes/n)o	7/25			4/11	3/14	0.6⁣^∗^
BMI (kg/m^2^)	26.8 (23.8–28.6)	24 (23.3–30.1)	0.7	27.1 (24.7–30.5)	25.5 (22.7–27.9)	0.2
WHR	0.9 (0.86–0.98)	0.9 (0.86–0.91)	0.4	0.91 (0.86–0.98)	0.93 (0.87–0.97)	0.97
Daily insulin dose (U/kg b.w./day)	0.46 (0.38–0.6)			0.42 (0.37–0.57)	0.56 (0.44–0.63)	0.08
MMSE	29 (27–29)	29 (28–30)	0.3	28 (26–29)	29 (28–30)	0.03^∗∗^
A1c (%)	7.95 (7.4–8.4)			8 (7.5–8.6)	7.9 (7.3–8.2)	0.5
TG (mmol/L)	1.28 (0.99–1.61)	1.29 (0.93–1.59)	0.98	1.38 (0.96–2.15)	1.15 (1.0–1.48)	0.4
TC (mmol/L)	4.94 (4.43–5.59)	4.75 (4.16–5.43)	0.7	4.97 (4.68–5.87)	4.91 (4.34–5.51)	0.98
LDL (mmol/L)	2.62 (2.03–3.12)	2.6 (1.95–3.26)	0.9	2.51 (1.34–3.07)	2.64 (2.25–3.18)	0.5
HDL (mmol/L)	1.68 (1.32–1.89)	1.38 (1.06–1.73)	0.2	1.78 (1.29–1.89)	1.68 (1.34–1.86)	0.9
TDI	31.6 (28.75–33.6)	34.1 (33.25–37.25)	0.02^∗∗^	31.5(26–33.75)⁣^∗∗∗∗^	32.5 (30–33.5)	0.04^∗∗∗^
Threshold	7.0 (6.5–8.0)	8.5 (8–9)	0.049^∗∗^	7.25 (6.5–8)	7 (6.5–8.0)	0.1
Differentiation	11 (9.5–13)	12 (12–13)	0.2	11 (9–12)	12 (11–13)	0.1
Identification	13 (11.5–14)	13 (13–15)	0.06	12 (10–14)	13 (12–14)	0.1
Summarized OB volume (mm^3^)	65.8 (57.95–71.7)	75.8 (74.8–76.7)	0.0005^∗∗^	58.1 (54.01–70.9)⁣^∗∗∗∗^	69.8 (65.02–72.2)⁣^∗∗∗∗^	0.0002^∗∗∗^
Left OB volume (mm^3^)	32.6 (29.1–35.4	37.95 (37.2–38.8)	0.0006^∗∗^	29.5 (27.1–35.2)⁣^∗∗∗∗^	34.6 (31.8–35.6)⁣^∗∗∗∗^	0.0002^∗∗∗^
Right OB volume (mm^3^)	33.4 (28.6–36.2)	37.5 (36.9–38.8)	0.001^∗∗^	29.3 (26.9–34.7)⁣^∗∗∗∗^	34.5 (32.7–36.7)	0.0006^∗∗∗^
Left PCo thickness (mm)	3.1 (2.8–3.4)	3.7 (3.6–4.1)	0.02^∗∗^	3.1 (2.7–3.4)⁣^∗∗∗∗^	3.1 (2.8–3.7)	0.03^∗∗∗^
Right PCo thickness (mm)	2.9 (2.7–3.4)	3.4 (3.2–3.9)	0.09	2.8 (2.6–2.98)	2.98 (2.7–3.6)	0.1

*Note:* Data are median (IQR) or *n*. Group comparisons: T1D versus controls and DPN versus No-DPN are performed using the Mann–Whitney test and or *χ*^2^ test. Olfactory test results and olfactory structure measurements were compared using the Mann–Whitney test (T1D vs. controls) and the Kruskall–Wallis test (controls vs. DPN vs. no-DPN). Post hoc analysis was performed with Dunn's test with *p* < 0.05. Statistically significant *p* values are shown in boldface.

Abbreviations: A1c, glycated hemoglobin A1c percentage; BMI, body mass index; CAN, cardiac autonomic neuropathy; DKD, diabetic kidney disease; DPN, diabetic peripheral neuropathy; DR, diabetic retinopathy; HDL, high-density lipoproteins cholesterol serum concentration; IQR, interquartile range; LDL, low-density lipoproteins cholesterol serum concentration; MMSE, Mini Mental State Examination; OB, olfactory bulb; PCo, piriform cortex; TC, total cholesterol serum concentration; TDI, threshold-differentiation-identification index; TG, triglycerides serum concentration; WHR, waist-hip ratio.

⁣^∗^*p* value for *χ*^2^ tests.

⁣^∗∗^*p* value for the Mann–Whitney test.

⁣^∗∗∗^*p* value for the Kruskal–Wallis test.

⁣^∗∗∗∗^Statistically significant difference with the controls for post hoc analysis with Dunn's test.

## Data Availability

Data is available on request through a data access committee, institutional review board, or the authors themselves—from Dr Maciej Chudzinski: maciej.a.chudzinski@gmail.com.

## Supporting Information

Supporting Information 1 Figure S1. Olfactory test results in patients with Type 1 diabetes with DPN and without DPN and in healthy controls. Abbreviations: TDI, threshold-differentiation-identification index; ns, not significant. Kruskall–Wallis test. *P*_interaction_ (*P*_int_) < 0.05 indicates that at least one subgroup result differs significantly. *p* value is represented by a diamond ♦ - *p* < 0.05.

Supporting Information 2 Table S1. Olfactory performance and rhinencephalon structures volumetry in patients with painful and painless DPN.

Supporting Information 3 Table S2. Correlations between structural measurements of the rhinencephalon and olfactory performance expressed as TDI.

Supporting Information 4 Table S3. Stepwise, multiple, linear (sigma-restricted) regression model for the OB summarized volume (*R*^2^ = 0.26; *p* = 0.03).

Supporting Information 5 Table S4. Effect sizes for Mann–Whitney test–Cliff's delta, for nonparametric comparisons of rhinencephalon structures and olfactory test results.

Supporting Information 6 Table S5. Equivalence tests for analyzed variables.
