# Supplementary figures and images for: Diabetic Neuropathy Is Related to Rhinencephalon Degeneration in Adults With Type 1 Diabetes
Source: J Diabetes Res. 2024 Oct 7;2024:6359972. doi: 10.1155/2024/6359972 (PMC11634408; doi:10.1155/2024/6359972)

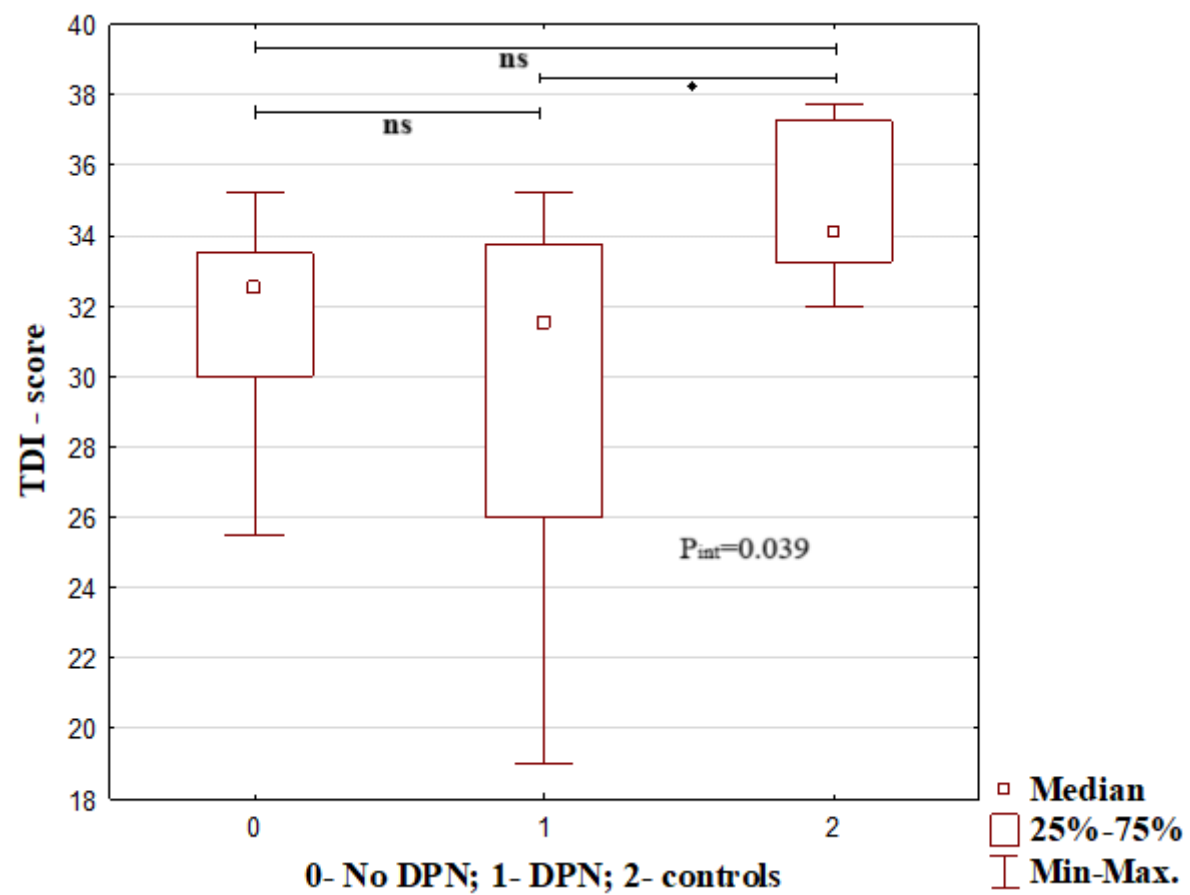

Supplement: Supporting Information 1 — Figure S1. Olfactory test results in patients with Type 1 diabetes with DPN and without DPN and in healthy controls. Abbreviations: TDI, threshold-differentiation-identification index; ns, not significant. Kruskall–Wallis test. Pinteraction (Pint) < 0.05 indicates that at least one subgroup result differs significantly. p value is represented by a diamond ♦ - p < 0.05. [file 6359972.f1.pdf]
