# Supplementary material for: Diabetic Neuropathy Is Related to Rhinencephalon Degeneration in Adults With Type 1 Diabetes
Source: J Diabetes Res. 2024 Oct 7;2024:6359972. doi: 10.1155/2024/6359972 (PMC11634408; doi:10.1155/2024/6359972)
Supplement: Supporting Information 2 — Table S1. Olfactory performance and rhinencephalon structures volumetry in patients with painful and painless DPN. [file 6359972.f2.doc]

**SUPPLEMENTARY TABLE 1** Olfactory performance and rhinencephalon structures volumetry in patients with painful and painless DPN.

| Variable | Patients with painful DPN n=8 | Patients with painless DPN n=7 | p |
| --- | --- | --- | --- |
| TDI | 32.4 (28.8-33.8) | 28.5 (23.5-34.5) | 0.5 |
| Threshold | 7.4 (6.6-8.5) | 7.25 (6-7.5) | 0.5 |
| Differentiation | 11(9.5-12) | 10 (8-13) | 1.0 |
| Identification | 13.5 (11-14.5) | 11 ( 9-13) | 0.1 |
| Summarized OB volume [mm^3^] | 57.8 (55-64.7) | 60.2 (49.8-70.9) | 0.7 |
| Left OB volume [mm^3^] | 29.1 (27.9-32.4) | 29.6 (25.2-35.2) | 1.0 |
| Right OB volume [mm^3^] | 28.3 (27.1-32) | 31.5 (24.6-35.7) | 0.6 |
| Left PCo thickness [mm] | 3.1 (2.7-3.3) | 3.3 (2.7-3.6) | 0.7 |
| Right PCo thickness [mm] | 2.7 (2.3-2.9) | 2.9 (2.7-3.3) | 0.1 |

Abbreviations: IQR, interquartile range; T1D, type 1 diabetes mellitus; DPN diabetic peripheral neuropathy; TDI threshold-differentiation-identification index; OB, olfactory bulb; PCo, pyriform cortex;

Data are median (IQR). Mann – Whitney test.
