# Supplementary material for: Diabetic Neuropathy Is Related to Rhinencephalon Degeneration in Adults With Type 1 Diabetes
Source: J Diabetes Res. 2024 Oct 7;2024:6359972. doi: 10.1155/2024/6359972 (PMC11634408; doi:10.1155/2024/6359972)
Supplement: Supporting Information 3 — Table S2. Correlations between structural measurements of the rhinencephalon and olfactory performance expressed as TDI. [file 6359972.f3.doc]

**SUPPLEMENTARY TABLE 2** Correlations between structural measurements of the rhinencephalon and olfactory performance expressed as TDI.

| Variables correlated with TDI | All participants n=38 | |
| --- | --- | --- |
|  | RS | p |
| Summarized OB volume [mm^3^] | 0.32 | 0.048 |
| Left OB volume [mm^3^] | 0.33 | 0.04 |
| Right OB volume [mm^3^] | 0.28 | 0.09 |
| Left PCo thickness [mm] | 0.43 | 0.006 |
| Right PCo thickness [mm] | 0.36 | 0.03 |

Abbreviations: TDI, threshold-differentiation-identification index; OB, olfactory bulb; PCo, pyriform cortex; RS, Spearman’s rank correlation coefficient.

Spearman’s rank correlation test.
