# Supplementary material for: Diabetic Neuropathy Is Related to Rhinencephalon Degeneration in Adults With Type 1 Diabetes
Source: J Diabetes Res. 2024 Oct 7;2024:6359972. doi: 10.1155/2024/6359972 (PMC11634408; doi:10.1155/2024/6359972)
Supplement: Supporting Information 4 — Table S3. Stepwise, multiple, linear (sigma-restricted) regression model for the OB summarized volume (R2 = 0.26; p = 0.03). [file 6359972.f4.doc]

**SUPPLEMENTARY TABLE 3** linear regression models for the OB summarized volume

| Predictors | coefficient β | p-value |
| --- | --- | --- |
| TDI | 0.53 | 0.177 |
| MMSE | 0.82 | 0.386 |
| A1c (%) | 0.55 | 0.619 |
| Sex (M=1) | -3.58 | 0.298 |
| Presence of DPN | -7.02 | 0.015 |
| Tobacco smoking | -7.89 | 0.013 |

Abbreviations: TDI, threshold-differentiation-identification index; MMSE, mini mental state examination; A1c, glycated hemoglobin A1c percentage; DPN, diabetic peripheral neuropathy.
