# Supplementary material for: Diabetic Neuropathy Is Related to Rhinencephalon Degeneration in Adults With Type 1 Diabetes
Source: J Diabetes Res. 2024 Oct 7;2024:6359972. doi: 10.1155/2024/6359972 (PMC11634408; doi:10.1155/2024/6359972)
Supplement: Supporting Information 5 — Table S4. Effect sizes for Mann–Whitney test–Cliff's delta, for nonparametric comparisons of rhinencephalon structures and olfactory test results. [file 6359972.f5.doc]

**SUPPLEMENTARY TABLE 4** Effect sizes for Mann-Whitney test – Cliff’s delta, for non-parametric comparisons of rhinencephalon structures and olfactory test results.

| Compared variables | Cliff’s delta | 95%CI lower | 95%CI upper |
| --- | --- | --- | --- |
| Summarized OB volumes in patients without DPN vs DPN | 0.51 | 0.09 | 0.77 |
| Summarized OB volumes in patients without DPN vs controls | -0.82 | -0.95 | -0.44 |
| Summarized OB volumes in patients with DPN vs controls | -1 | -1 | -0.97 |
| Right PCo thickness in patients without DPN vs DPN | 0.33 | -0.08 | 0.65 |
| Right PCo thickness in patients without DPN vs controls | -0.31 | -0.71 | 0.23 |
| Right PCo thickness in patients with DPN vs controls | -0.61 | -0.88 | -0.03 |
| Left PCo thickness in patients without DPN vs DPN | 0.19 | -0.22 | 0.55 |
| Left PCo thickness in patients without DPN vs controls | -0.48 | -0.78 | 0.01 |
| Left PCo thickness in patients with DPN vs controls | -0.80 | -0.99 | 0.25 |
| Summarized OB volumes in patients with T1D vs controls | -0.91 | -0.97 | -0.68 |
| Right PCo thickness in patients with T1D vs controls | -0.45 | -0.77 | 0.03 |
| Left PCo thickness in patients with T1D vs controls | -0.63 | -0.85 | -0.21 |
