# Supplementary material for: Diabetic Neuropathy Is Related to Rhinencephalon Degeneration in Adults With Type 1 Diabetes
Source: J Diabetes Res. 2024 Oct 7;2024:6359972. doi: 10.1155/2024/6359972 (PMC11634408; doi:10.1155/2024/6359972)
Supplement: Supporting Information 6 — Table S5. Equivalence tests for analyzed variables. [file 6359972.f6.doc]

**SUPPLEMENTARY TABLE 5.** Equivalence tests for analyzed variables

| Compared variables | Delta -75% of H-L estimate* | H0: Delta - theta <= 0 | H0: theta + Delta <= 0 |
| --- | --- | --- | --- |
| Summarized OB volumes in patients without DPN vs DPN | 6.8 | 0.9853 | 0.0035 |
| Summarized OB volumes in patients without DPN vs controls | 4.6 | 0.9956 | 0.0006 |
| Summarized OB volumes in patients with DPN vs controls | 12.8 | 0.9939 | <0.0001 |
| Right PCo thickness in patients without DPN vs DPN | 0.21 | 0.9450 | 0.0533 |
| Right PCo thickness in patients without DPN vs controls | 0.26 | 0.8651 | 0.1271 |
| Right PCo thickness in patients with DPN vs controls | 0.46 | 0.9824 | 0.0147 |
| Left PCo thickness in patients without DPN vs DPN | 0.11 | 0.8216 | 0.1762 |
| Left PCo thickness in patients without DPN vs controls | 0.34 | 0.9550 | 0.0407 |
| Left PCo thickness in patients with DPN vs controls | 0.51 | 0.9972 | 0.0022 |
| Summarized OB volumes in patients with T1D vs controls | 7.4 | 0.9993 | 0.0001 |
| Right PCo thickness in patients with T1D vs controls | 0.34 | 0.9581 | 0.0395 |
| Left PCo thickness in patients with T1D vs controls | 0.44 | 0.9919 | 0.0073 |

* Hodges-Lehmann shift estimate
